# Membrane-dependent relief of translation elongation arrest on pseudouridine- and *N*^1^-methyl-pseudouridine-modified mRNAs

**DOI:** 10.1093/nar/gkab1241

**Published:** 2021-12-22

**Authors:** Yuri V Svitkin, Anne-Claude Gingras, Nahum Sonenberg

**Affiliations:** Department of Biochemistry, McGill University, Montréal, Québec H3A 1A3, Canada; Rosalind and Morris Goodman Cancer Institute, Montréal, Québec H3A 1A3, Canada; Lunenfeld-Tanenbaum Research Institute, Sinai Health System, and Department of Molecular Genetics, University of Toronto, Toronto, Ontario, M5G 1×5, Canada; Department of Biochemistry, McGill University, Montréal, Québec H3A 1A3, Canada; Rosalind and Morris Goodman Cancer Institute, Montréal, Québec H3A 1A3, Canada

## Abstract

Expression of therapeutically important proteins has benefited dramatically from the advent of chemically modified mRNAs that feature decreased lability and immunogenicity. This had a momentous effect on the rapid development of COVID-19 mRNA vaccines. Incorporation of the naturally occurring pseudouridine (Ψ) or *N*^1^-methyl-pseudouridine (N1mΨ) into *in vitro* transcribed mRNAs prevents the activation of unwanted immune responses by blocking eIF2α phosphorylation, which inhibits translation. Here, we report that Ψs in luciferase (Luc) mRNA exacerbate translation pausing in nuclease-untreated rabbit reticulocyte lysate (uRRL) and promote the formation of high-order-ribosome structures. The major deceleration of elongation occurs at the Ψ-rich nucleotides 1294–1326 of Ψ-Luc mRNA and results in premature termination of translation. The impairment of translation is mainly due to the shortage of membranous components. Supplementing uRRL with canine microsomal membranes (CMMs) relaxes the impediments to ribosome movement, resolves collided ribosomes, and greatly enhances full-size luciferase production. CMMs also strongly stimulated an extremely inefficient translation of N1mΨ-Luc mRNA in uRRL. Evidence is presented that translational pausing can promote membrane recruitment of polysomes with nascent polypeptides that lack a signal sequence. Our results highlight an underappreciated role of membrane binding to polysomes in the prevention of ribosome collision and premature release of nascent polypeptides.

## Graphical Abstract

**Graphical Abstract:**
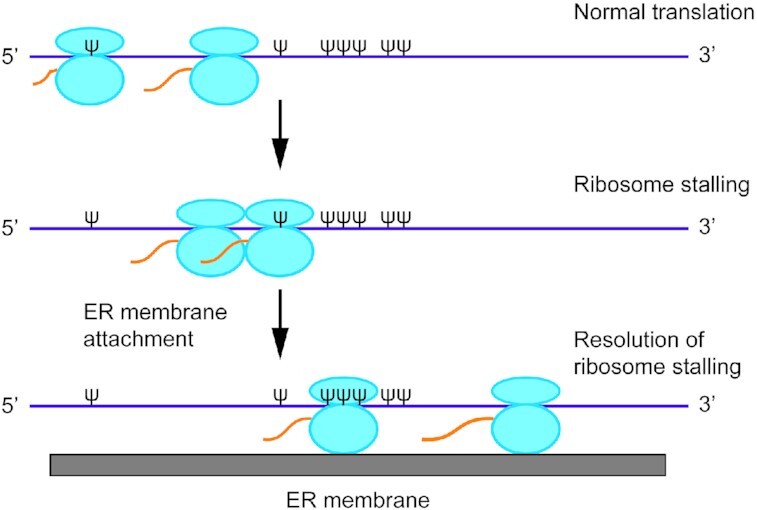
Translational pausing on ψ or *N*^1^mψ-containing mRNAs promotes membrane recruitment.

## INTRODUCTION

*In vitro* transcribed mRNAs are safer and more efficient for gene delivery than viral vectors or plasmid DNA as demonstrated in fundamental and pre-clinical studies (1). The major therapeutic applications of mRNA include immunotherapy against infectious diseases and cancer, protein replacement, gene editing, and regenerative medicine. Of these applications, the mRNA vaccine field developed most rapidly (2). For example, two doses of SARS-CoV-2 spike protein mRNA-based vaccines against the pandemic respiratory illness COVID-19 developed by Moderna and Pfizer/BioNTech were found to induce a robust neutralizing antibody response as well as CD4+ and CD8+ T-cell responses in humans (3,4). Furthermore, these vaccines have proven to be nearly 95% effective in preventing COVID-19 (5,6).

The problem of instability and immunogenicity of mRNA, which initially precluded its therapeutic use, has been largely solved by incorporating natural base modifications, such as pseudouridine (Ψ), *N*^1^-methyl-pseudouridine (N1mΨ), 5-methyl cytosine (5mC), *N*^6^-methyl adenosine (m6A) and 2-thiouridine (s2U), into mRNA (1,7–10).

Chemically modified nucleosides in mRNA prevent the activation of several RNA sensors including interferon-induced 2′-5′-oligoadenylate synthetases (OAS). OAS produces short 2′–5′-linked oligomers (2–5A) that trigger RNAse L-mediated cleavage of single-stranded RNA (11). mRNA containing modified nucleosides, such as Ψ and m6A, only poorly activates OAS and therefore does not induce the production of 2–5A (12). In addition, the substitution of N1mΨs or 5-methoxy-uridines for Us induces global changes in mRNA secondary structure and engenders high protein expression in cells by increasing mRNA half-life (13).

Also, replacing of uridines with Ψ or s2U abrogates stimulation of Toll-like receptors (TLRs) and retinoic acid-inducible gene I protein (RIG-I) by mRNA (7). Most importantly, modified nucleosides in mRNA lessen innate antiviral response by preventing the activation of RNA-dependent protein kinase (PKR) (14,15). PKR, among other cellular functions, represses translation under conditions of cellular stress or virus infection by phosphorylating the α-subunit of translation initiation factor 2 (eIF2α) (16,17). eIF2, which is composed of three subunits α, β and γ, delivers methionyl initiator tRNA (Met-tRNA_i_) to the 40S ribosomal subunit in the form of a ternary complex with GTP and Met-tRNA_i_. After GTP hydrolysis, the eIF2-associated GDP is exchanged for GTP by the nucleotide exchange factor eIF2B. Phosphorylation of eIF2α alters eIF2, so that it binds more tightly to eIF2B, thus inhibiting the recycling of eIF2-GDP to the active GTP-bound form and attenuating global translation. Since eIF2 is more abundant than eIF2B, even low amounts of phosphorylated eIF2α are sufficient to cause a block in eIF2B activity (17). PKR is activated by double-stranded RNA, which is generated during virus infection, in a process that requires dimerization and autophosphorylation of the kinase. However, single-stranded RNA containing stable secondary structures or aberrant products of *in vitro* transcription can also activate PKR (14,15,18,19). Others and we previously reported that the translational enhancement imparted by Ψ, N1mΨ, and other nucleoside modifications in mRNA can be recapitulated in several cell-free extracts and demonstrated that this stimulation is primarily due to reduced activation of PKR (15,20).

Most studies on the modulation of translation by modified nucleosides in mRNA have been focused on the initiation step, which is under most circumstances rate-limiting (21,22). However, modified nucleosides can also alter the rate of polypeptide elongation and ultimately affect overall protein expression. It is well established that ribosomes move along mRNAs with uneven speed (23–26). The slowdown of elongation and ribosome pausing is dictated by mRNA features, such as stretches of rare codons, the availability of corresponding aminoacyl-tRNAs, translation factors, the amino acid composition of the nascent protein, and high order RNA structures. Although ribosome pausing plays an important role in the folding of nascent polypeptides and promotes protein-protein interactions, it may also lead to ribosome collisions with co-translational degradation of both mRNA and the nascent chain (25,26). We previously observed that N1mΨ, either alone or in combination with other nucleoside modifications, impedes ribosome movement at defined sites on modified mRNAs (20). Here we investigated in greater detail the effect of global substitution of Ψ and N1mΨ for U in luciferase (Luc) mRNA on polypeptide chain elongation. We chose these models because Ψ is the most common natural nucleoside modification, and because Ψ and N1mΨ are known to confer the highest translation enhancement to mRNA in most mammalian cells, cell-free extracts, and mice (8,20,27–31). We demonstrate that incorporating Ψ into Luc mRNA while enhancing the translational capacity of the mRNA in HEK293T cells and Krebs cell-free extracts, causes elongation arrest upon translation in a micrococcal nuclease (MN)-untreated rabbit reticulocyte lysate (uRRL). The presence of N1mΨ in Luc mRNA is also detrimental for elongation in this system. We found unexpectedly that the addition of rough endoplasmic reticulum (ER)-derived microsomal vesicles to the stalled ribosomal complexes relieves the translational arrest.

## MATERIALS AND METHODS

### mRNA preparation

Ψ, N1mΨ and 5mC triphosphates were obtained from TriLink Biotechnologies. Unmodified or Ψ, N1mΨ, 5mC and Ψ/5mC-nucleoside modified polyadenylated (A_96_) Luc mRNAs were prepared by T3 polymerase *in vitro* transcription of BamHI-linearized T3luc(A)+ plasmid (32). Overlap extension PCR was used to delete nucleotides 1294–1326 or 1294–1317 in the Luc open reading frame, and the final purified PCR product was used for *in vitro* transcription. mRNAs were purified by RNase-free DNase (Roche) treatment, phenol-chloroform extraction, 2 M LiCl precipitation, and centrifugation through CHROMA SPIN-200 columns (Clontech). All mRNAs were capped using the Vaccinia enzyme ScriptCap m^7^G capping system (CELLSCRIPT) and further purified by phenol–chloroform extraction and ethanol precipitation. For quality assurance, the mRNA preparations were analyzed by denaturing agarose gel electrophoresis.

### mRNA transfection

Human embryonic kidney (HEK293T) cells were maintained in Dulbecco's modified Eagle's medium (DMEM) containing 2 mM l-glutamine, 10% fetal calf serum (FBS), 100 U/ml penicillin, and 100 μg/ml streptomycin. One day before transfection, the cells were seeded into 96-well plates at a density of 6 × 10^4^ cells/well. Unmodified or nucleoside-modified mRNAs (90 ng) were transfected into ∼90% confluent cells using a TransIT-mRNA transfection kit as recommended by the manufacturer (Mirus). After culturing for 18 h, cells were lysed in 100 μl of Passive lysis buffer (Promega) with a single freeze-thaw cycle. Lysates were clarified by centrifugation. Aliquots (12 μl) of the 100-fold diluted samples were assayed for luciferase activity using the Luciferase assay system (Promega) and Lumat LB 9507 bioluminometer (Berthold Technologies).

### *In vitro* translation assays

The preparation of translation-competent S10 extract from Krebs-2 ascites carcinoma cells was described previously (33,34). Translation of Luc mRNAs was carried out using standard techniques (33). MN-treated RRL (Promega) was used as described by the manufacturer. uRRL (Promega) was used as described (35). Reaction mixtures (12.5 μl), in the presence or absence of mRNA (4 μg/ml), were incubated at 30°C for 1 h or for the times indicated in the figure legends. Canine microsomal membranes (CMMs) or CMM buffer (Promega) were included in the reaction mixtures where indicated. Reactions were stopped by a 30-fold dilution with PBS containing 0.6 mM cycloheximide. Luciferase activity was measured in 3-μl aliquots of samples supplemented with 9 μl of Reporter lysis buffer (Promega). For Western blot analysis of luciferase synthesis, the samples were supplemented with four reaction volumes of SDS-sample buffer. Nascent polypeptides were released from peptidyl-tRNAs by ribonuclease A (RNase A) or puromycin treatment (36). For RNase A treatment, aliquots of the reaction mixtures were supplemented with two volumes of buffer A (50 mM Tris–HCl, pH 6.8, 10 mM EDTA and 2% SDS). After the addition of RNase A to 100 μg/ml final concentration and incubation at 37°C for 10 min, the samples were fixed with SDS-sample buffer. For puromycin treatment, aliquots of the reactions were supplemented with puromycin (1.8 mM final concentration), incubated at 30°C for 20 min, and terminated with SDS-sample buffer. For protease protection assay, the translation reactions (12.5 μl) containing uRRL, CMMs (7.2% by volume), and either unmodified or Ψ-incorporated Luc mRNAs (0.1 μg) were incubated at 30°C for 90 min. After translation, 0.2 M CaCl_2_ was added to the samples at a final concentration of 10 mM. The samples were then incubated with proteinase K (100 μg/ml) in the absence or presence of 1% Triton X-100 at 4°C for 60 min. Reactions were stopped by the addition of 0.75 μl of 0.1 M PMSF in isopropanol and immediately transferred to SDS-PAGE sample buffer that was preheated to 98°C (37).

### Analysis of nascent polypeptides

A previously described protocol with few modifications was followed (38,39). Unmodified and Ψ- or N1mΨ-containing Luc mRNAs were translated in MN-treated RRL (Promega) in the presence of [^35^S]methionine as recommended by the manufacturer. CMMs (7.2% by volume) or control buffer were present where indicated. Reaction mixtures (100 μl), in the presence or absence of mRNA (4 μg/ml), were incubated at 30°C for 20 min. After incubation, the samples were diluted with an equal volume of buffer B (10 mM Tris–HCl, pH 7.6, 100 mM KCl, 10 mM MgCl_2_) containing 1% Triton X-100, laid on top of 450 μl of 30% glycerol in buffer B, and centrifuged for 1 h at 4°C and 100 000 × *g* in TLA-120.2 rotor (Beckman Coulter). The polysome pellets were suspended in 18 μl of 0.5 mg/ml RNase A solution in 1 mM Tris–HCl, pH 7.6, and incubated at 37°C for 30 min. To facilitate the hydrolysis of peptidyl–tRNA ester bonds, NaOH (0.1 M) was added to a final concentration of 10 mM, and the incubation was continued for an additional 30 min. After the addition of the SDS-sample buffer, the nascent polypeptides were resolved using SDS-15% PAGE. Gels were fixed, treated with EN^3^HANCE (PerkinElmer), dried, and subjected to fluorography at –70°C.

### Western blotting

Luciferase proteins from *in vitro* translation samples were resolved by SDS–12% PAGE, transferred onto a nitrocellulose membrane, and detected using Western Lightning chemiluminescence kit (PerkinElmer). The primary rabbit monoclonal antibody directed against the N-terminal 1–100 amino acid region of Firefly Luciferase (EPR17789) was obtained from Abcam (ab185923) and used diluted 1:4000. The secondary HRP-conjugated anti-rabbit antibody (GE Healthcare) was used diluted 1:10 000.

### Analysis of polysome profiles

Unmodified or Ψ-incorporated Luc mRNAs were radiolabeled at the poly(A) tail using [α-^32^P]ATP and yeast poly(A) polymerase (20,40). ^32^P-poly(A)-labeled mRNAs (∼2 × 10^6^ cpm, 200 ng) were incubated in a total reaction volume of 50 μl with uRRL at 30°C for 15 min. For analysis of 80S initiation complex formation, the reaction mixtures were supplemented with 0.6 mM cycloheximide and incubated at 30°C for 5 min. Reactions were stopped by 5-fold dilution with ice-cold polysome (P) buffer (15 mM Tris–HCl, pH 7.5, 15 mM Mg(OAc)_2_, 0.3 M NaCl and 0.2 mg/ml heparin) containing 0.6 mM cycloheximide and 0.2% Triton X-100. Ribosomal complexes were resolved by centrifugation (Beckman SW41 rotor, 37 000 rpm, 2 h, and 4°C) through 7.5–45% sucrose gradients prepared with buffer P. Fractions (0.36 ml) were collected manually from the top of the gradients, and radioactivity was measured by liquid scintillation counting. Radioactivity in each fraction was presented as a percentage of total radioactivity recovered from the gradient.

### Analysis of mRNA stability

uRRLs (100 μl) were incubated with unmodified or Ψ-incorporated Luc mRNAs (4 μg/ml) at 30°C. At the indicated times, 15-μl aliquots of the reaction mixtures were withdrawn and supplemented with 200 μl of SDS/proteinase K solution (41). After incubation for 15 min at room temperature, total RNA was extracted with phenol-chloroform and precipitated with ethanol. RNA was resolved by formaldehyde–1% agarose gel electrophoresis and transferred onto nylon membranes (Hybond-N, GE Healthcare). To confirm that equal amounts of total RNA were loaded in each lane, the blots were stained with Blot Stain Blue (Sigma) and the intensities of bands of 28S ribosomal RNA (rRNA) were measured using NIH Image J software. RNA was then hybridized with a 44 nt-long ^32^P-labeled DNA oligo complementary to the Luc mRNA coding region using ExpressHyb hybridization solution (Clontech Laboratories, Inc), as described by the manufacturer. The blots were exposed to X-ray films. Bands of Luc mRNA were quantified using a Typhoon PhosphorImager (GE Healthcare).

### Membrane binding assay of Luc and Ψ-Luc mRNA

Unmodified or Ψ-incorporated Luc mRNAs were ^32^P-labeled on their poly(A) tails as described above. The mRNAs (∼2.5 × 10^5^ cpm, 25 ng) were subjected to *in vitro* translation in MN-treated RRL (50 μl) with or without CMMs (3.6 μl). After incubation at 30°C for 12 min, 7.5 μl (15%) aliquots of the reaction mixtures were withdrawn for analysis of mRNA integrity (input). The remaining portions of the samples were layered onto 100 μl of 1.2 M sucrose cushions in buffer D (25 mM HEPES–KOH, pH 7.3, 50 mM KCl, 75 mM KOAc, 2 mM MgCl_2_) (33) and centrifuged for 30 min at 4°C and 20 000 × *g* to sediment microsomal membranes and associated polysomes. After centrifugation and careful removal of the supernatants, the pellet fractions were suspended in 200 μl of SDS/proteinase K solution. Total RNA from input and pellet fractions was isolated as described above. After separation by formaldehyde–1% agarose gel electrophoresis and transfer onto nylon membranes, the radiolabeled RNAs were detected by autoradiography.

## RESULTS

### Translation elongation arrest on the Ψ-containing Luc mRNA in uRRL

**Figure 1.**
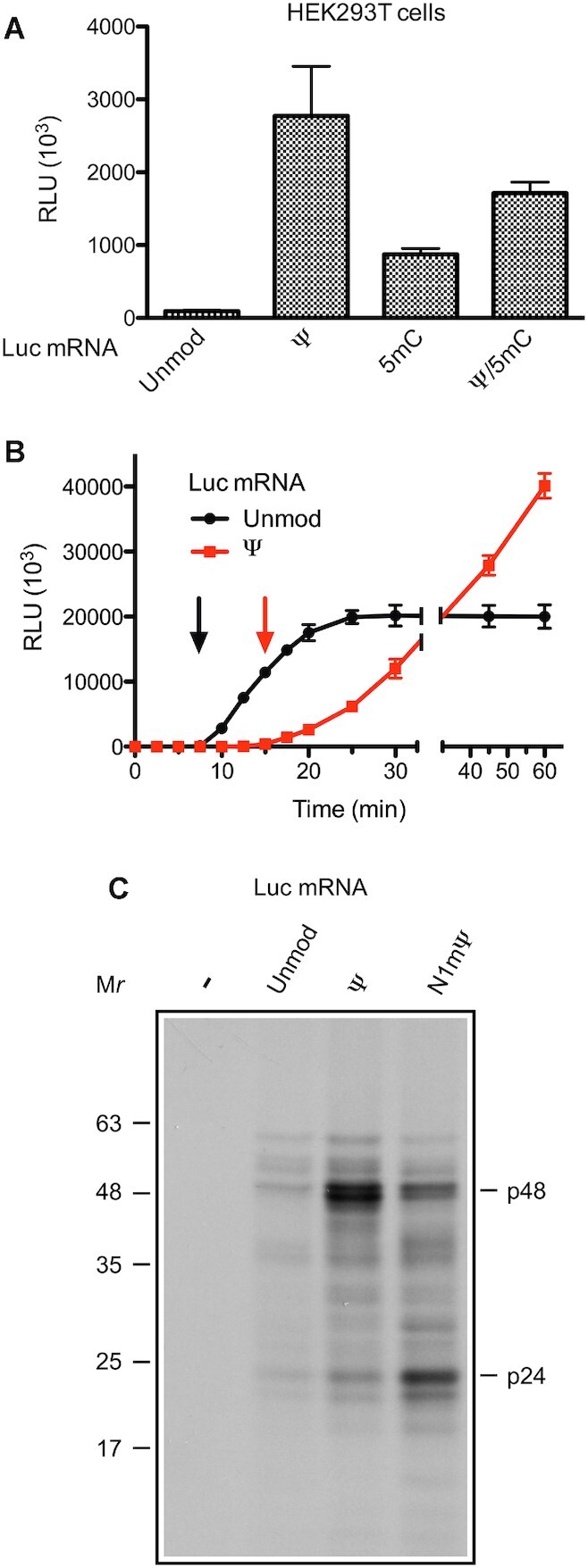
Translational enhancement of Luc mRNA by Ψ nucleoside modifications. (**A**) Comparative effects of Ψ, 5mC, and 5mC/ Ψ nucleoside modifications in Luc mRNA on its translation in cells. Luc mRNAs, unmodified (Unmod) or containing the indicated nucleoside modifications, were transfected into HEK293T cells. Cells were lysed 18 h after transfection and luciferase activity was measured in 1% aliquots of the lysates. (**B**) Time course of luciferase synthesis in MN-treated RRL translating unmodified (red) or Ψ-incorporated (black) Luc mRNAs. At the indicated time points, 1-μl aliquots of the reaction mixtures were assayed for luciferase activity. Arrows in matching colors indicate the time points of first appearance of luciferase activity (7.5 and 15 min after the beginning of translation of unmodified and Ψ-containing Luc mRNAs, respectively). The mean values from four (A) or three (B) independent assays ± SD are shown. RLU, relative luciferase units. (**C**) ^35^S-labeled nascent polypeptides accumulating during translation of the indicated mRNAs in MN-treated RRL were isolated and analyzed as described in Materials and Methods. In all lanes, equal aliquots were subjected to analysis. The major stalled polypeptides (p48 and p24) and molecular weight markers are indicated.

*In vitro* transcribed mRNAs in which all uridines are replaced by Ψ or N1mΨ exhibit very high translation efficiency and low immunogenicity, thus holding significant promise for protein replacement therapies (12,15,20,31). Figure 1A illustrates the superior translation capacity of a Ψ-modified mRNA in cultured cells. In this experiment, Luc mRNAs, either lacking or containing one (Ψ or 5mC), or two (Ψ/5mC) types of modified nucleosides, were transfected in HEK293T cells and luciferase activity was monitored 18 h post-transfection. All the modified mRNAs produced higher amounts of luciferase than the standard Luc mRNA (Figure 1A). Importantly, the Ψ nucleoside modification elicited the strongest stimulation of luciferase synthesis (29-fold) as compared to the 5mC (9-fold) or Ψ/5mC combination (18-fold). Thus, extensive mRNA modification may not be required for optimal protein expression.

Studies of reporter expression in cell-free systems derived from mammalian cells and bacteria resulted in conflicting conclusions regarding the impact of Ψs in mRNA on translation. While some studies suggested that the presence of Ψs enhances translation (8,15), others reported inhibition of protein synthesis (42,43). To reconcile these observations, we compared the translation of unmodified or Ψ-incorporated Luc mRNA in a MN-treated RRL. Consistent with earlier results (15), the Ψ-Luc mRNA yielded ∼2-fold more luciferase than Luc mRNA upon incubation for 60 min with MN-treated RRL (Figure 1B, Supplementary Figure S1). Notably, the translation of Ψ-Luc mRNA, but not Luc mRNA, did not level off by 25 min of incubation, consistent with the inability of Ψ-Luc mRNA to activate PKR and suppress translation initiation (15). However, luciferase activity was first detected at a later time after the beginning of translation of Ψ-Luc than Luc mRNA (15 min vs. 7.5 min). Consequently, the Ψ-Luc mRNA underperformed Luc mRNA at the early time points (<30 min). Incorporation of N1mΨs into Luc mRNA also extended the duration of its first round of translation in MN-treated RRL, which has been attributed to the reduction of elongation velocity (20). Therefore, it is likely that Ψs in Luc mRNA, while being beneficiary for translation initiation and luciferase expression over the long term, reduces the rate of elongation in MN-treated RRL.

To further buttress this notion, we isolated nascent polypeptides accumulating on ribosomes after translation of unmodified or Ψ- and N1mΨ-containing Luc mRNAs in MN-treated RRL in the presence of [^35^S]methionine. This was done by centrifugation of the reaction mixtures through a layer of 30% glycerol solution. Nascent polypeptides in polysomal pellets were then treated with ribonuclease A, resolved by SDS PAGE, and detected by fluorography. The interpretation of the results was based on the fact that nascent polypeptides when synthesized at a slow rate are overrepresented and appear as discrete bands (38). With this technique, we revealed two major pausing sites on Ψ-Luc and N1mΨ-Luc mRNAs, as their translation yielded two prominent nascent polypeptides, p48 and p24 (represented by duplets of closely positioned bands) (Figure 1C). Interestingly, for an unexplained reason, the levels of pausing at these sites were different for Ψ-Luc and N1mΨ-Luc mRNAs. Particularly, p48 appeared as the predominant product of Ψ-Luc mRNA translation but was synthesized less efficiently than p24 from the N1mΨ-Luc mRNA. In contrast, the unmodified Luc mRNA produced only weak and not well-resolved bands, indicating its largely uniform mode of translation. Notably, no full-size luciferase protein was evident because of its release from polysomes after synthesis. In addition, no proteins were detected in the absence of mRNA (negative control). Obviously, the observed ribosome pauses are largely transitory, as they do not compromise the superior performance of Ψ-Luc and N1mΨ-Luc mRNAs in MN-treated RRL (Supplementary Figure S1).

**Figure 2.**
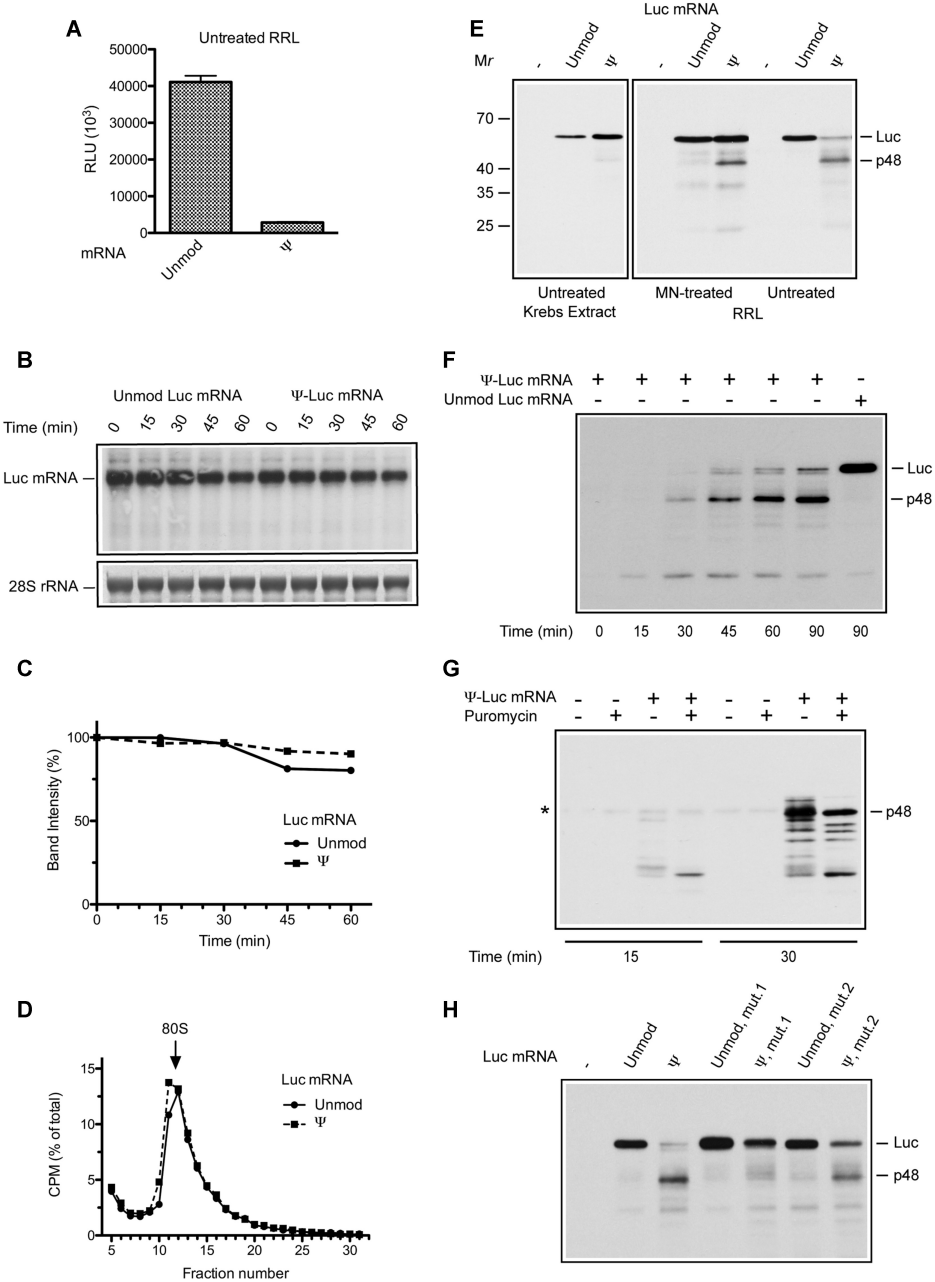
Translation elongation arrest on the Ψ-Luc mRNA in uRRL. (**A**) Luciferase synthesis in uRRL programmed with unmodified or Ψ-containing Luc mRNA. The RLU values reported are for 1-μl aliquots of the reaction mixtures. (**B**) Unmodified Luc and Ψ-Luc mRNAs are stable in uRRL. The mRNAs were programmed into uRRL at a final concentration of 4 μg/ml. Total RNA was isolated at the indicated times from aliquots of the reaction mixtures, and Luc mRNA integrity was analyzed by Northern blotting (top panel). 28S ribosomal RNA (rRNA) visualized by staining was used as a loading control (bottom panel). (**C**) Quantifications of Luc mRNA signals from panel B. Band intensities at time 0 were set as 100%. (**D**) 80S initiation complex formation on ^32^P-labeled Luc and Ψ-Luc mRNA in cycloheximide-supplemented uRRL. After incubation at 30°C for 5 min, the reaction mixtures were analyzed by sucrose gradient centrifugation as described in Materials and Methods. Four top fractions of the gradients are not shown for greater clarity. (**E**) Western blot analysis of luciferase polypeptides produced by Luc or Ψ-Luc mRNAs upon translation in untreated Krebs extract, MN-treated RRL, and uRRL. In lanes (-) no mRNA was added. The positions of full-length (Luc) and truncated (p48) luciferase polypeptides and molecular mass markers are indicated. (**F**) Time course of protein synthesis in Ψ-Luc mRNA-programmed uRRL. Ψ-Luc mRNA was translated under standard reaction conditions. At the indicated times, aliquots of the reaction mixture were withdrawn and analyzed by Western blotting. The products of translation of unmodified Luc mRNA are shown for comparison. (**G**) uRRL was incubated with or without Ψ-Luc mRNAs. At the indicated times, aliquots of the reaction mixtures were withdrawn and either treated or untreated with puromycin as described in Materials and Methods. The analysis of luciferase polypeptides was as described for panel E. (**H**) Unmodified or Ψ-containing Luc mRNA either without or with deletions of nucleotides 1294–1326 (mut.1) and 1294–1305 (mut.2) were translated in uRRL, and the reaction mixtures were analyzed by western blotting. In panel G, asterisk indicates a nonspecific band that migrates slightly slower than p48 and is present in all the lanes including the minus mRNA control lane.

We next compared the translation of unmodified and Ψ-containing Luc mRNA in nuclease-untreated RRL (uRRL), which mimics the physiological conditions of mRNA competition. Strikingly and unexpectedly, the presence of Ψs in Luc mRNA almost completely abolished its translation in uRRL (∼17-fold inhibition at 60 min compared to unmodified mRNA) (Figure 2A). Thus uRRL roughly resembles bacterial systems with respect to inefficient translation of Ψ-containing mRNAs (42). The very low yields of luciferase could be due to several reasons including degradation of mRNA, inefficient translation initiation, and insufficiency of a cognate aminoacylated tRNA. We first sought to determine whether the Ψ nucleoside modifications destabilize the Luc mRNA in uRRL. Northern blot analysis of Luc and Ψ-Luc mRNA decay showed that both mRNAs are stable in this system, with more than 80% of the mRNA remaining intact after 60 min of incubation (Figure 2B and C). Thus, the extremely weak performance of the Ψ-Luc mRNA in uRRL is not due to its degradation. To address the possibility of inhibition of translation initiation, we compared the efficiencies of 80S initiation complex formation on ^32^P-labeled Ψ-Luc and Luc mRNAs. The mRNAs were used to program uRRL in the presence of cycloheximide for a short time (5 min), and the 80S complexes were resolved from the unbound mRNAs by sucrose gradient centrifugation. The analysis did not reveal a decrease in the rate of 80S initiation complex formation with the Ψ-containing Luc mRNA (Figure 2D).

Next, we examined the possibility of premature termination of translation. To this end, the Ψ-Luc and Luc mRNAs were programmed into uRRL, and their translation products were analyzed by western blotting using an antibody against the N-terminal region of luciferase. While after 60 min, the translation of unmodified Luc mRNA gave rise to a major product of ∼61 kDa corresponding to the full-size luciferase, the Ψ-Luc mRNA was predominantly translated into a truncated ∼48 kDa polypeptide (p48) appearing as a somewhat diffuse band (Figure 2E). Occasionally, some smaller products of the Ψ-Luc mRNA translation were also visible. The full-size luciferase band became more distinct after a longer (90 min) translation of the Ψ-Luc mRNA, as revealed by the time-course analysis of protein synthesis (Figure 2F). However, it was still much less intense than that produced by the translation of unmodified Luc mRNA. Notably, p48 was resistant to RNase A or puromycin treatments and is, therefore, a terminal product of translation that lacks covalently attached tRNA (Supplementary Figure S2 and Figure 2G). Overall, it is likely that one major and multiple minor translation pausing processes converge to inhibit the complete translation of the Ψ-Luc in uRRL.

Based on the size of the aberrant p48 product, we hypothesized that the Ψ-rich sequence (45% Ψs) spanning nucleotides 1294–1326 of the Ψ-Luc mRNA open reading frame is the site of translation termination (Supplementary Figure S3). The presence of Ψ in mRNA codons has been reported to impede ribosome movement (44). To confirm the assignment of the major termination site, we deleted nucleotides 1294–1326 from the Ψ-Luc mRNA. In agreement, this deletion prevented the accumulation of p48, while increasing the yield of the full-size protein (Figure 2H, compare lanes Ψ and Ψ, mut.1). Inhibition of p48 synthesis was also observed after deletion of a smaller segment, nucleotides 1294–1317, of the Ψ-Luc mRNA (Figure 2H, compare lanes Ψ and Ψ, mut.2). However, this inhibition was only partial indicating the contribution of the 3′-terminal sequence Ψ_1318_CΨΨΨAAΨΨ_1326_ to translational termination. Notably, both deletions failed to completely rescue the yield of the full-size luciferase. This is most likely because they do not nullify other, less prominent, ribosome stalling events on the Ψ-Luc mRNA.

Interestingly, in MN-treated RRL, the majority of ribosomes overcome the translation impediments on the Ψ-Luc mRNA, although synthesis of the p48 protein was also apparent (Figure 2E). This suggests that ribosome stalling on Ψ-Luc mRNA is exaggerated under conditions of mRNA competition in uRRL. Significantly, in Krebs extract, the absence of nuclease treatment did not affect uninterrupted translation of Ψ-Luc mRNA, which was markedly higher than Luc mRNA (Supplementary Figure S4 and Figure 2E). Also, this system showed no evidence of p48 formation upon the translation of Ψ-Luc mRNA.

### Membranes relieve translation elongation arrest on Ψ-Luc mRNA

**Figure 3.**
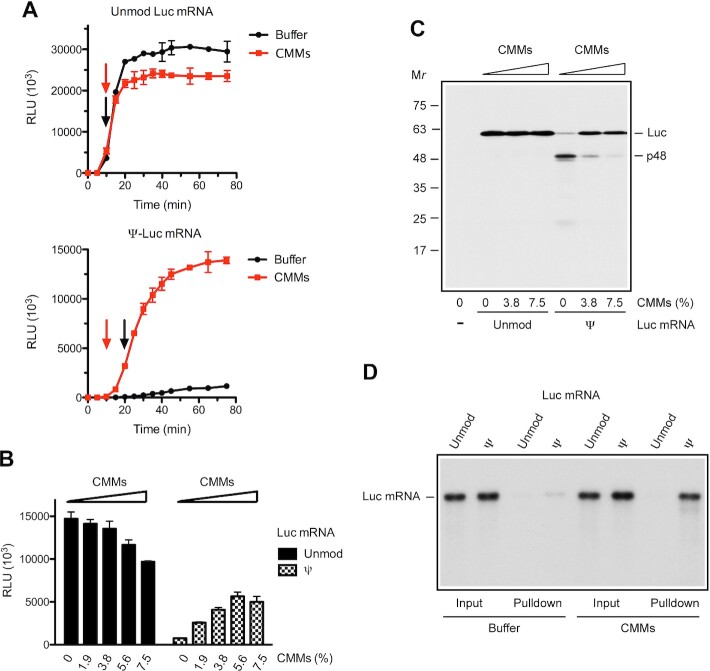
Membranes relax elongation impediments on the Ψ-Luc mRNA in uRRL. (**A**) Kinetics of luciferase synthesis in uRRL programmed with unmodified (top) or Ψ-containing (bottom) Luc mRNAs, in the absence (black) or presence (red) of CMMs (used at 6% of the total reaction volume). One microliter aliquots of the reaction mixtures were withdrawn at the indicated times and assayed for luciferase activity. Arrows in matching colors indicate the time points of first appearance of luciferase activity. (**B**) Unmodified or Ψ-containing Luc mRNAs were translated in uRRL in the presence of the indicated amounts of CMMs. The mean values from three (A) or four (B) independent assays ± SD are shown. (**C**) The products of translation from panel B were analyzed by Western blotting. In lane (–) no mRNA was added. (**D**) Membrane-binding assay of unmodified and Ψ-modified Luc mRNAs. The indicated ^32^P-labeled mRNAs were incubated with MN-treated RRL in the presence of CMMs or control buffer as described in Materials and Methods. After the withdrawal of 15%-aliquots of the reaction mixtures for RNA integrity check (input), the samples were centrifuged to sediment microsomal membranes containing associated mRNAs. RNAs isolated from the input and pulldown fractions were analyzed by agarose gel electrophoresis and autoradiography.

We next sought conditions that would improve the processivity of the Ψ-Luc mRNA translation in uRRL. It is conceivable that uRRL lacks a component(s) that is present in Krebs extract to facilitate elongation. This component might be rough ER-derived microsomal membrane vesicles, which are abundant in extracts of nucleated cells (45). We thus investigated the effect of commercially available Canine Microsomal Membranes (CMMs) on the time course of luciferase synthesis directed by the Ψ-Luc mRNA in uRRL. While being ineffective and somewhat inhibitory for the translation of the unmodified Luc mRNA, CMMs dramatically enhanced (∼12-fold at 75 min) luciferase synthesis from the Ψ-Luc mRNA (Figure 3A). Furthermore, consistent with the acceleration of elongation, CMMs shortened the delay in the first appearance of luciferase activity from 20 to 10 min in the Ψ-Luc mRNA-programmed reaction. In contrast, CMMs did not expedite the appearance of luciferase activity in the course of the translation of the unmodified Luc mRNA. The increased yields of the full-size luciferase in the Ψ-Luc mRNA-programmed uRRL were CMMs dose-dependent and occurred at the expense of p48 production (Figure 3B and C). We conclude that targeting the ribosome-nascent chain complex (RNC) to the ER membrane plays a critical role in the processivity of Ψ-Luc mRNA translation.

To investigate whether membrane binding is specific for polysomes translating Ψ-Luc mRNA, we programmed ^32^P-labeled Ψ-Luc and Luc mRNA into uRRL in the presence of CMMs. The membrane fraction was recovered by centrifugation, and mRNA isolated thereof was analyzed by agarose gel electrophoresis. Ψ-Luc mRNA was enriched in the membrane fraction, while Luc mRNA was not (Figure 3D). Importantly in the absence of CMMs, almost no Ψ-Luc or Luc mRNA was detected in the pellet fraction. We surmise that the stalled Ψ-Luc mRNA-RNC carries a signal for membrane attachment. Notwithstanding the membrane localization of Ψ-Luc mRNA, the luciferase polypeptide that was produced by its translation was not imported into the ER lumen as indicated by its sensitivity to the digestion with proteinase K (Supplementary Figure S5).

### Membranes resolve ribosome collisions on the Ψ-Luc mRNA

**Figure 4.**
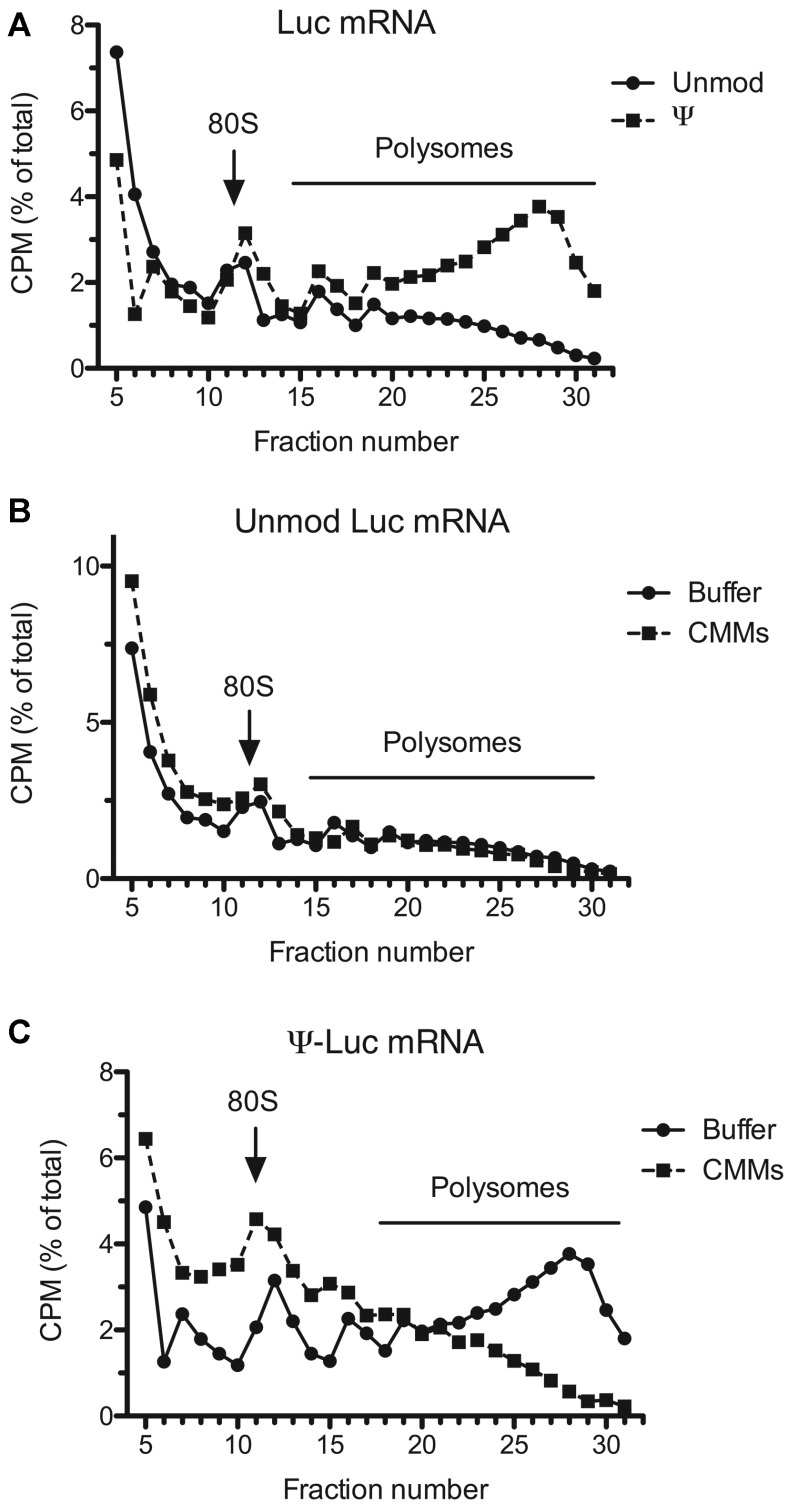
Membrane-dependent resolution of collided ribosome structures on the Ψ-incorporated Luc mRNA in uRRL. (**A**) uRRLs were incubated at 30°C for 15 min with ^32^P-labeled Luc or Ψ-Luc mRNA. Polysomes were analyzed by sucrose gradient centrifugation and counting of radioactivity in the collected fractions. Consistent with elongation impediments, a much higher proportion of Ψ-containing Luc mRNA was detected in polysome fractions as compared to unmodified Luc mRNA. (**B**) ^32^P-labeled Luc mRNA was translated in uRRLs supplemented with CMMs or control buffer. The presence of CMMs had little or no effect on the polysome profile of unmodified Luc mRNA. (**C**) ^32^P-labeled Ψ-Luc mRNA was translated in uRRLs supplemented with CMMs or control buffer. The presence of CMMs drastically reduced the proportion of Ψ-incorporated Luc mRNA in polysome fractions, indicating resolution of the stalled ribosome complexes. In (B) and (C), the relative volume of the CMMs fraction was 5.6%. Other conditions were as in (A). Radioactivity in each fraction is presented as a percentage of total counts recovered from the gradient. Four top fractions of the gradients are omitted for greater clarity.

Site-specific stalling of ribosomes on the Ψ-Luc mRNA can result in ribosome collision, which would be expected to increase polysome size and abundance. Ribosome collision occurs when a trailing ribosome bumps into a slow-moving leading ribosome (46). To test this prediction, we examined the polysomal profiles of ^32^P-labeled Luc and Ψ-Luc mRNAs in uRRL after 15 min incubation at 30°C. Consistent with the translational pausing on Ψ-Luc mRNA, its association with heavy ribosomal complexes, i.e., those containing more than three ribosomes, was much more pronounced than that of Luc mRNA (Figure 4A). Strikingly, the addition of CMMs to the Ψ-Luc mRNA-programmed reaction mixtures dissociated the high-order ribosome complexes, while not decreasing the complexity of polysomes translating unmodified Luc mRNA (Figure 4B and C). Thus, ribosome pausing and stacking during translation of the Ψ-Luc mRNA in uRRL could be well explained by the deficiency of membranes in this system.

### Membrane-dependent rescue of N1mΨ-Luc mRNA translation in uRRL

**Figure 5.**
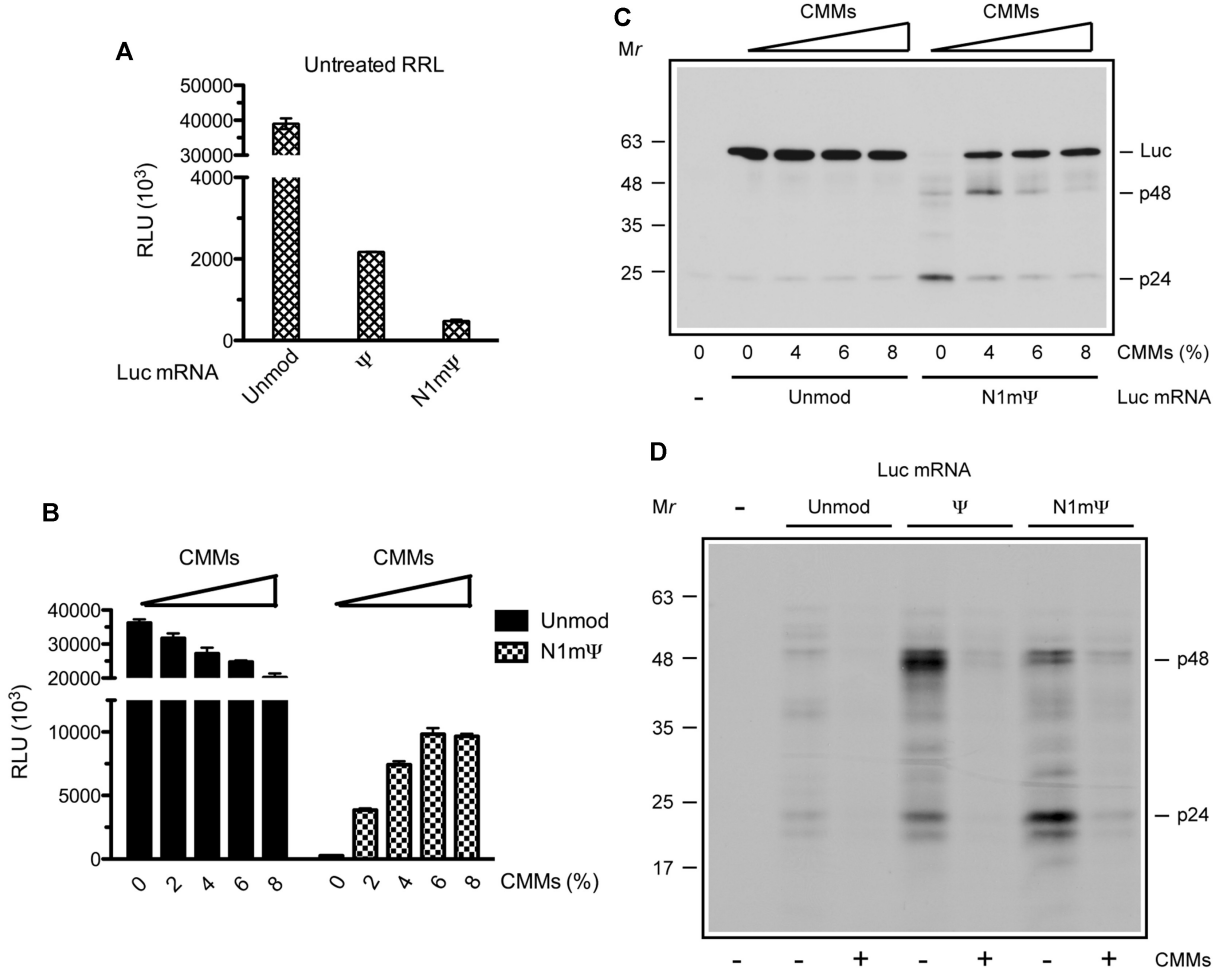
Membranes relieve translation elongation arrest on the N1mΨ-Luc mRNA in uRRL. (**A**) Luciferase synthesis in uRRL programmed with unmodified or Ψ- and N1mΨ-modified Luc mRNAs. (**B**) CMM dose responses of translation of unmodified and N1mΨ-modified Luc mRNAs in uRRL. The relative volumes of the CMMs fraction are indicated. Incubation was at 30°C for 60 min (A) or 90 min (B). Luciferase activity was measured in 1-μl aliquots of the reaction mixtures. The mean values from three (A) or four (B) independent assays ± SD are shown. (**C**) The products of translation from panel B were analyzed by Western blotting. The positions of the major translation products and molecular weight markers are indicated. (**D**) ^35^S-labeled nascent polypeptides accumulating during translation of the indicated mRNAs in MN-treated RRL in the absence or presence of CMMs were isolated and analyzed as described in Materials and Methods. In all lanes, equal aliquots were subjected to analysis. The major stalled polypeptides (p48 and p24) and molecular mass markers are indicated.

The N1mΨ nucleoside modification is currently used in the generation of COVID-19 vaccines, as known to confer superior stability and performance to cell-transfected mRNA (13,20,31,47). Consistent with this, in MN-treated RRL and Krebs extract, the N1mΨ-Luc mRNA outperformed both unmodified and Ψ-containing Luc mRNA (Supplementary Figure S1) (20). However, the N1mΨ alteration of Luc mRNA also engendered ribosome pausing and reduced the rate of polypeptide elongation in this system (Figure 1C) (20). We reasoned that the conditions of translation in uRRL would enforce ribosome stalling on the N1mΨ-Luc mRNA, thus enabling us to study this phenomenon in more detail. Indeed, the N1mΨ-Luc mRNA-directed luciferase synthesis was dramatically decreased in uRRL (Figure 5A). The reduction of active luciferase output by N1mΨ was even stronger than that by Ψ (81- versus 18-fold).

Next, we analyzed the ability of CMMs to rescue the N1mΨ-Luc mRNA translation in uRRL. Thus, we added CMMs in increasing concentrations to uRRL and performed translation assays using unmodified or N1mΨ-modified Luc mRNAs. For the N1mΨ-Luc mRNA, we observed a strong stimulation of luciferase synthesis after CMM addition (up to 36-fold), whereas the unmodified mRNA translation was markedly inhibited by CMMs (Figure 5B). An interesting nuance had been that in the absence of CMMs, the termination of translation on the N1mΨ-Luc mRNA occurred earlier than on the Ψ-Luc mRNA, as evidenced by the predominant accumulation of the p24 polypeptide (Figure 5C). The robust p48 production, indicating the advancement of ribosomes to the downstream nucleotide 1294–1326 translation block, was only detected after the addition of a small amount of CMMs to the reaction mixtures (4% of the reaction volume). Under these conditions, a sizeable amount of the full-size luciferase protein was also produced. Importantly, CMMs fully rectified the aberrant translation pattern of the N1mΨ-Luc mRNA when used at the optimal concentration (8% of the reaction volume).

We also investigated the ability of CMMs to avert ribosome stalling by ^35^S-labeling and analysis of nascent polypeptides produced during the translation of Ψ-Luc and N1mΨ-Luc mRNAs in MN-treated RRL. In the absence of CMMs, both mRNAs directed robust incorporation of [^35^S]methionine into p48 and p24 nascent polypeptides (Figure 5D). Other, less intense, bands were also apparent. Importantly, when CMMs were present, the accumulation of p48, p24, and other stalled polypeptides was dramatically reduced. As expected, the translation of unmodified Luc mRNA did not yield prominent paused products. These results suggest that CMMs act to smoothen overall elongation rates on Ψ-Luc and N1mΨ-Luc mRNAs.

## DISCUSSION

Nucleoside modifications in cell-transfected mRNAs suppress the activation of PKR, resulting in low eIF2α phosphorylation and enhanced translation initiation (15). In addition, nucleoside modifications can impact other steps of translation. Previously, we observed the delayed appearance of luciferase activity in several *in vitro* systems programmed with nucleoside-modified Luc mRNAs and explained this phenomenon by the deceleration of polypeptide chain elongation (20). Here, we describe an extreme case, in which the Ψ nucleotide-rich sequence in position 1294–1326 of the Ψ-Luc mRNA engenders translation blockage and termination. Thus, under conditions of translation in the uRRL, the Ψ-Luc mRNA directs predominantly synthesis of a C-terminally truncated polypeptide (p48) and several smaller products rather than full-size luciferase. The translation of the N1mΨ-containing Luc mRNA in uRRL is also incomplete, but in this case, with the predominant formation of a smaller prematurely terminated product, p24. Consistent with the importance of the C-terminal 12 amino acids for luciferase activity, both p48 and p24 proteins are enzymatically inactive (48).

The reason why the translation of the Ψ-Luc mRNA in uRRL stops prematurely is not immediately clear, but cleavage of the mRNA can be ruled out as the cause. The most likely scenario is that the Ψ nucleotide-rich sequences are decoded at a slower rate than unmodified sequences and that the elongation deceleration threshold necessary for nascent peptide release is reached at nucleotide positions 1294–1326. In eukaryotes, translation termination is initiated by recognition of a stop codon in the ribosomal A site by a release factor, eRF1 (24,49). This highly specific interaction typically prevents the translation termination at sense codons. However, the presence of Ψ in mRNA codons or ribosome stalling may weaken this specificity (50,51). Another possibility is that ribosome collisions induce + 1 frameshifting, similar to that observed in yeast and bacteria (52). Within the 1294–1326 nucleotide segment, this would result in translation termination at one of three termination codons, Ψ_1301_AG, Ψ_1313_GA and Ψ_1322_AA (Supplementary Figure S3). Of these, the Ψ_1322_AA codon appears to be most relevant since the deletion of nucleotides 1294–1317 from Ψ-Luc mRNA resulted in a reduction, but not an elimination of p48 formation. As mentioned above, the presence of N1mΨ in Luc mRNA causes ribosome stalling at a site upstream of the 1294–1317 nucleotide position and the premature release of p24 in uRRL. Precise location and identification of nucleotide sequence or structure that slows ribosome progression and prematurely terminates translation on the N1mΨ-Luc mRNA awaits further experimentation. In MN-treated RRL, the pause sites on Ψ-Luc and N1mΨ-Luc mRNAs are largely overcome by translating ribosomes. It is plausible that the cessation of translation of the Ψ/N1mΨ-Luc mRNA in uRRL results from the competition with endogenous mRNAs for components of the translation machinery. This competition would be expected to decrease the availability of aminoacyl-tRNA, aminoacyl-tRNA synthetases, elongation factors, and other components necessary for the translation of problematic RNA sequences.

A major consequence of ribosome pausing during translation of Ψ-Luc and N1mΨ-Luc mRNAs is the formation of high-order-ribosome complexes, presumably representing collided ribosomes (Figure 4), (20). Since Ψ can stabilize RNA duplexes (53,54), slow decoding of the Ψ-rich codons could result from the decreased dissociation rate of tRNA from the ribosomal E-site. In favor of this hypothesis is the demonstration that the presence of three tRNAs on the ribosome slows translation elongation rate in a prokaryotic system (55). A poor translation of multiple Ψ-containing mRNAs was found to occur in wheat germ and bacterial translation systems (42,43). Furthermore, replacing even a single U with Ψ in an mRNA codon impedes amino acid addition and EF-Tu GTPase activation in bacterial systems (42,44). Similarly, the presence of m6A and 2′ O-methyl-containing codons in mRNA could alter aminoacyl-tRNA binding and accommodation (56,57). Another possible explanation is that the substitution of Ψ or N1mΨ for U in Luc mRNA slows ribosome processivity by stabilizing RNA secondary structure (58,59). Increased secondary structure stability has been reported for Luc mRNA containing the N1mΨ or 5-methoxy-uridine modifications (13). The stabilization of specific stem-loop structures in this mRNA by Ψs is also possible (54). The stable mRNA stem-loops could pause the ribosome by hindering the ribosomal A-site tRNA binding, as recently reported for a bacterial cell-free system (60).

In yeast, ribosome collision has been shown to trigger ribosome-associated quality control involving among other processes extraction of the ribosome-stalled mRNA by the Ski complex and its subsequent degradation via the exosome (61–63). However, in uRRL ribosome stalling on the Ψ-Luc mRNA does not accelerate mRNA decay at least during the first hour of incubation (Figure 2B and C). This may be due to the lack of the quality control sensor ZNF598 in RRL that recognizes a unique interface between the collided ribosomes (46).

An intriguing finding in this study is that polysomes translating Ψ-Luc mRNA, but not Luc mRNA, are recruited to the membranes in CMMs-supplemented RRL (Figure 3D). This interaction decreases ribosome pausing, resolves heavy polysomes and promotes the complete translation of Ψ-Luc mRNA (Figures 3B, C and 4). Likewise, CMMs dramatically improved the translation of N1mΨ-Luc mRNA in uRRL (Figure 5). It is unclear whether the Ψ/N1mΨ-Luc mRNA translating ribosomes are anchored to the membrane by the nascent polypeptides or directly contacting the membrane surface. Secretory and membrane proteins possess stretches of hydrophobic amino acids (either a cleavable N-terminal signal sequence or a transmembrane domain) that target the mRNA-RNC to the ER membrane via the signal recognition particle (SRP)-SRP receptor pathway (64–67). In this pathway, the SRP binds to the hydrophobic domain of the nascent polypeptide and slows its elongation. The SRP–mRNA–RNC complex is recruited to the ER via the interaction between SRP and the SRP receptor. This interaction releases the SRP from the RNC and offsets the elongation pausing. The RNC complex is then delivered to the SEC61 translocon that serves both as a ribosome receptor and translocation channel (68). Luciferase lacks the N-terminal signal sequence. However, the SRP may recognize some signals in elongation-arrested luciferase moieties that are translated from the Ψ/N1mΨ-Luc mRNA. In contrast, the fast translation of the unmodified Luc mRNA might not provide sufficient time for SRP to complete the targeting reaction due to a rapid sequestering of the signal sequences with the growing polypeptide chain. The requirement for translation pausing has been shown for the SRP-mediated recruitment of the unspliced X-box-binding protein 1 (XBP1u) mRNA to the membranes (36,69). In addition, membrane targeting was dependent on the presence of an internal hydrophobic region in XBP1u (HR2). Significantly, like luciferase, most XBP1u was not imported into the ER lumen during translation, presumably because of insufficient hydrophobicity of HR2 (69,70). Relatedly, in yeast, the local slowdown of translation by nonoptimal codons clusters has been reported to promote the recognition of nascent polypeptide chains by SRP (71). Overall, these results point to the role of translational pausing in extending the competent state at which SRP can recognize an exposed signal sequence in the nascent polypeptide chain.

An alternative and perhaps more likely possibility is that the Ψ/N1mΨ-Luc mRNA-RNC uses an SRP-independent mechanism for membrane targeting (72,73). Indeed, it has been known for a long time that the large ribosomal subunit possesses a site of membrane attachment (74–76). Furthermore, evidence was presented for the Sec61 translocon-independent ribosome binding (77,78). In this process, LRRC59 and some other integral membrane proteins have been proposed to play the role of ribosome receptors (72,79,80). An attractive hypothesis would be that the stalled ribosomal complexes, such as those formed on the Ψ/N1mΨ–Luc mRNA during translation, provide the signals for membrane attachment. A salient feature of collided ribosomes (disomes) is the unique 40S-40S interface, which could be become bound by the ubiquitin ligase ZNF598 (46,81). In addition, colliding ribosomes can recruit the mitogen-activated protein kinase kinase kinase (MAPKKK) ZAKα, resulting in its activation by auto-phosphorylation (82). It is possible that the 40S–40S interface is also recognized by some integral membrane protein(s). Whatever the mechanism of membrane attachment to the Ψ/N1mΨ–Luc mRNA–RNC complex might be, it is clear that the local slowdown of translation is a prerequisite for this process.

Ribosomes stall, collide, and form queues as a consequence of stochastic translation (23,83,84). For example, in mouse liver and budding yeast, the ribosome queuing rate has been estimated to reach 10% and 20%, respectively (85). Ribosome collisions are known to be more frequent under a variety of stress conditions (82). In light of our data, the widespread occurrence of elongation stalls could be the reason why so many mRNAs, not just those encoding secretory and membrane proteins, are translated on the surface of the ER membrane (72,77,78,86–90).

It is unclear how CMMs relieve ribosome pauses and collisions on the Ψ/N1mΨ–Luc mRNA. A plausible explanation would be that the attachment of polysomes to membranes smoothens the overall elongation rate by accelerating excessively slow and/or decelerating excessively fast ribosomal movement. So far no evidence for the role of membranes in securing spacing between translating ribosomes has been obtained. However, it is noteworthy that in mammalian cells the rate of protein synthesis on ER-bound ribosomes is 2.5–4-fold higher than that on cytosolic ribosomes, as indicated by *in situ*^35^S-Met/Cys pulse-chase labeling studies of ribosome-associated nascent peptide chains (91). This difference in translation rate could be a consequence of the divergent regulation of the tRNA aminoacylation/deacylation cycle in the cytosol and ER compartments (91). In addition, the ribosome binding integral membrane protein LRRC59 or other factors might positively regulate the ER-localized mRNA translation (80). Presumably, the membrane-dependent increase in translation rate largely removes elongation barriers on the Ψ/N1mΨ-Luc mRNA in uRRL. It is noteworthy that the complete translation of some long unmodified mRNAs *in vitro* may also benefit from the additions of CMMs, as we showed previously for hepatitis C virus (HCV) RNA (41). Further experiments will be required to dissect the mechanisms by which the addition of CMMs (and potentially other membrane preparations) relieves ribosomal collisions.

Strikingly, in contrast to translation in untreated Krebs extract, the translation of Ψ-Luc and N1mΨ–Luc mRNAs in uRRL was less efficient than Luc mRNA even in the presence of CMMs. It is thus likely that there is as yet an unidentified factor in the extracts of nucleated cells that enhances the overall translation rate of Ψ-Luc and N1mΨ–Luc mRNAs on top of the contribution from ER-derived microsomal vesicles. One candidate factor is the ASC-1 complex, which disassembles collided and ZNF598-ubiquitinated ribosomes (92). Both the ASC-1 complex and ZNF598 are deficient in RRL.

Our results are of broad significance given the pervasive presence of Ψ nucleotide modifications across mammalian transcriptomes (27–30). Significantly, the vast majority of Ψ residues in mRNA are located in coding regions (44). Ribosome pausing at the Ψ-containing sites may fine-tune the folding of the nascent polypeptide chains thereby enhancing the output of functional proteins. In this respect, the Ψ nucleotides in mRNA may functionally cooperate with other factors, such as synonymous codons, RNA secondary structure, and amino acid composition of nascent polypeptides in the modulation of local elongation rate, and ultimately protein production, folding, and co-translational assembly of protein complexes (25,59,93–97). In addition, the Ψ-mediated pausing of ribosomes could provide a signal for membrane recruitment to ameliorate the deleterious effects of random ribosome collisions and regulate diverse cellular processes. Ψ-mediated translational control could be particularly relevant under stress conditions that alter mRNA pseudouridylation (27,29,30,98). Furthermore, the potential targeting of synthetic Ψ and N1mΨ nucleoside-modified mRNAs to the ER membrane should be considered and studied in the course of the development of mRNA-based vaccines and therapeutics.

## NOTE ADDED IN PROOFS

Since the submission of the manuscript it has become clear that a third dose of mRNA vaccines is required to obtain maximum protection against SARS-CoV-2.

## DATA AVAILABILITY

All data generated and analyzed in this study are included in the manuscript and supplementary information.

## Supplementary Material

gkab1241_Supplemental_File
