# Supplementary material for: Membrane-dependent relief of translation elongation arrest on pseudouridine- and N1-methyl-pseudouridine-modified mRNAs
Source: Nucleic Acids Res. 2021 Dec 22;50(13):7202–15. doi: 10.1093/nar/gkab1241 (PMC9303281; doi:10.1093/nar/gkab1241)
Supplement: gkab1241_Supplemental_File [file gkab1241_supplemental_file.pdf]

Membrane-dependent relief of translation elongation arrest on pseudouridine- and N[1]-methyl-pseudouridine-modified mRNAs

Yuri V. Svitkin,[1,2,*] Anne-Claude Gingras,[3] and Nahum Sonenberg[1,2,*]

[1]Department of Biochemistry, McGill University, Montréal, Québec, H3A 1A3, Canada
[2]Rosalind and Morris Goodman Cancer Institute, Montréal, Québec, H3A 1A3, Canada
[3]Lunenfeld-Tanenbaum Research Institute, Sinai Health System, and Department of Molecular Genetics, University of Toronto, Toronto, Ontario, M5G 1X5, Canada

* To whom correspondence should be addressed.
Tel: +1 514 398 7274; Fax: +1 514 398 1286; Email: nahum.sonenberg@mcgill.ca or
Tel: +1 514 398 5695, Fax: +1 514 398 1286; Email: yuri.svitkine@mcgill.ca.

SUPPLEMENTARY DATA

Contents:

Supplementary Figures S1-S5

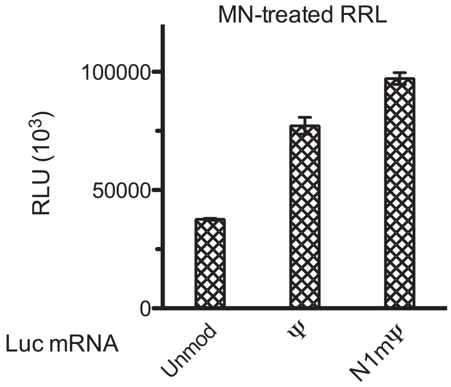

Figure S1. Ψ and N1mΨ nucleoside modifications in Luc mRNA enhance translation in MN-untreated RRL. Unmodified Luc, Ψ-Luc, and N1mΨ-Luc mRNA (4 µg/ml) were translated at 30°C for 1 h. One µl aliquots of the reaction mixtures were assayed for luciferase activity. The mean values of the triplicate data ± SD are shown.

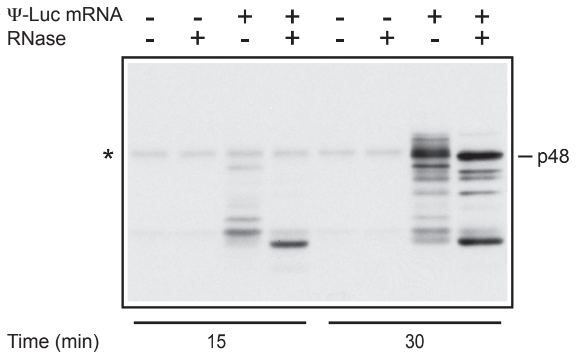

Figure S2. uRRL was incubated with or without Ψ-Luc mRNAs at 30°C for 15 or 30 min. Buffer A was then added to stop the reactions. *In vitro* translation products were further treated with RNAse A or left untreated. Western blot analysis of luciferase polypeptides was as described in Materials and methods. The position of the major prematurely terminated polypeptide (p48) is indicated. Asterisk indicates a nonspecific band that migrates slightly slower than p48 and is present in all the lanes including the minus mRNA control lanes.

AUGGAAGACGCCAAAAACAUAAAGAAAGGCCCGGCGCCAUUCUAUCCUCUAGAGGAUGGA 60
ACCGCUGGAGAGCAACUGCAUAAGGCUAUGAAGAGAUACGCCCUGGUUCCUGGAACAAUU 120
GCUUUUACAGAUGCACAUAUCGAGGUGAACAUCACGUACGCGGAAUACUUCGAAAUGUCC 180
GUUCGGUUGGCAGAAGCUAUGAAACGAUAUGGGCUGAAUACAAAUCACAGAAUCGUCGUG 240
UGCAGUGAAAACUCUCUUCAAUUCUUUAUGCCGGUGUUGGGCGCGUUAUUUAUCGGAGUU 300
GCAGUUGCGCCCGCGAACGACAUUUAUAAUGAACGUGAAUUGCUCAACAGUAUGAACAUU 360
UCGCAGCCUACCGUAGUGUUUGUUUCCAAAAAGGGGUUGCAAAAAAUUUUGAACGUGCAA 420
AAAAAAUUACCAAUAAUCCAGAAAAUUAUUAUCAUGGAUUCUAAAACGGAUUACCAGGGA 480
UUUCAGUCGAUGUACACGUUCGUCACAUCUCAUCUACCUCCCGGUUUUAAUGAAUACGAU 540
UUUGUACCAGAGUCCUUUGAUCGUGACAAAACAAUUGCACUGAUAAUGAAUUCCUCUGGA 600
UCUACUGGGUCACCUAAGGGUGUGGCCCUUCCGCAUAGAACUGCCUGCGUCAGAUUCUCG 660
CAUGCCAGAGAUCCUAUUUUUGGCAAUCAAAUCAUUCCGGAUACUGCGAUUUUAAGUGUU 720
GUUCCAUUCCAUCACGGUUUUGGAAUGUUUACUACACUCGGAUAUUUGAUAUGUGGAUUU 780
CGAGUCGUCUUAAUGUAUAGAUUUGAAGAAGAGCUGUUUUUACGAUCCCUUCAGGAUUAC 840
AAAAUUCAAAGUGCGUUGCUAGUACCAACCCUAUUUUCAUUCUUCGCCAAAAGCACUCUG 900
AUUGACAAAUACGAUUUAUCUAAUUUACACGAAAUUGCUUCUGGGGGCGCACCUCUUUCG 960
AAAGAAGUCGGGGAAGCGGUUGCAAAACGCUUCCAUCUUCCAGGGAUACGACAAGGAUAU 1020
GGGCUCACUGAGACUACAUCAGCUAUUCUGAUUACACCCGAGGGGGAUGAUAAACCGGGC 1080
GCGGUCGGUAAAGUUGUUCCAUUUUUUGAAGCGAAGGUUGUGGAUCUGGAUACCGGGAAA 1140
ACGCUGGGCGUUAAUCAGAGAGGCGAAUUAUGUGUCAGAGGACCUAUGAUUAUGUCCGGU 1200
UAUGUAAACGAUCCGGAAGCGACCAACGCCUUGAUUGACAAGGAUGGAUGGCUACAUUCU 1260
GGAGACAUAGCUUACUGGGACGAAGACGAACAC*UUCUUCAUAGUUGACCGCUUGAAGUCU* 1320
*U**UAA**UU*AAAUACAAAGGAUGUCAGGUGGCCCCCGCUGAAUUGGAAUCGAUAUUGUUACAA 1380
CACCCCAACAUCUUCGACGCGGGCGUGGCAGGUCUUCCCGGCGAUGACGCCGGUGAACUU 1440
CCCGCCGCCGUUGUUGUUUUGGAGCACGGAAAGACGAUGACGGAAAAAGAGAUCGUGGAU 1500
UACGUCGCCAGUCAAGUAACAACCGCGAAAAAGUUGCGCGGAGGAGUUGUGUUUGUGGAC 1560
GAAGUACCGAAAGGUCUUACCGGAAAACUCGACGCAAGAAAAAUCAGAGAGAUCCUCAUA 1620
AAGGCCAAGAAGGGCGGAAAGUCCAAAUUGUAA 1653

Figure S3. The coding sequence of Luc mRNA. The site of elongation arrest at the Ψ-modified U-rich sequence (nucleotides 1294-1326) is in italic. The sequence of nucleotides 1294-1317, which partially inhibits elongation, is underlined. The Ψ-modified U_{1322}AA codon, which is the potential termination codon in the case of +1 frameshifting, is in bold italic.

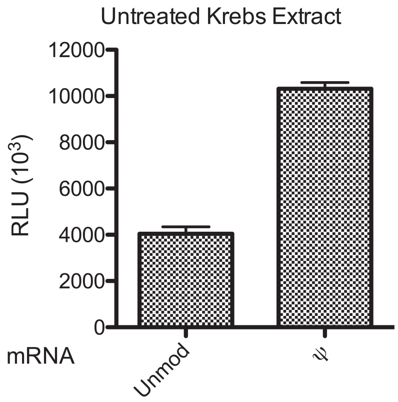

Figure S4. Enhancement of translation by Ψ-nucleoside modifications in Luc mRNA in MN-untreated Krebs extract. The extracts were incubated with Luc or Ψ-Luc mRNA (4 µg/ml) at 30°C for 1 h. One µl aliquots of the reaction mixtures were assayed for luciferase activity. The mean values of the triplicate data ± SD are shown.

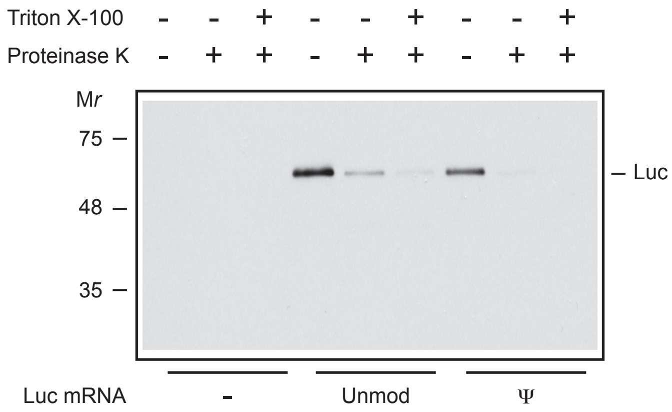

Figure S5. Protease protection assay. uRRL was supplemented with CMMs and incubated with or without unmodified Luc or Ψ-Luc mRNAs at 30°C for 60 min. The reaction products were then treated with proteinase K in the absence or presence of 1% Triton X-100 or left untreated. Western blot analysis of luciferase polypeptides was as described in Materials and methods.
